# Functional magnetic resonance imaging can be used to explore tactile and nociceptive processing in the infant brain

**DOI:** 10.1111/apa.12848

**Published:** 2014-12-01

**Authors:** Gemma Williams, Lorenzo Fabrizi, Judith Meek, Deborah Jackson, Irene Tracey, Nicola Robertson, Rebeccah Slater, Maria Fitzgerald

**Affiliations:** 1 UCL Department of Neuroscience, Physiology & Pharmacology, University College LondonLondon, UK; 2 Elizabeth Garrett Anderson Obstetric Wing, University College HospitalLondon, UK; 3 Nuffield Department of Clinical Neurosciences, Oxford Centre for Functional Magnetic Resonance Imaging of the Brain (FMRIB), University of OxfordOxford, UK

**Keywords:** Brain, Cutaneous stimulation, Magnetic resonance imaging, Neonatal, Pain

## Abstract

**Aim:**

Despite the importance of neonatal skin stimulation, little is known about activation of the newborn human infant brain by sensory stimulation of the skin. We carried out functional magnetic resonance imaging (fMRI) to assess the feasibility of measuring brain activation to a range of mechanical stimuli applied to the skin of neonatal infants.

**Methods:**

We studied 19 term infants with a mean age of 13 days. Brain activation was measured in response to brushing, von Frey hair (vFh) punctate stimulation and, in one case, nontissue damaging pinprick stimulation of the plantar surface of the foot. Initial whole brain analysis was followed by region of interest analysis of specific brain areas.

**Results:**

Distinct patterns of functional brain activation were evoked by brush and vFh punctate stimulation, which were reduced, but still present, under chloral hydrate sedation. Brain activation increased with increasing stimulus intensity. The feasibility of using pinprick stimulation in fMRI studies was established in one unsedated healthy full-term infant.

**Conclusion:**

Distinct brain activity patterns can be measured in response to different modalities and intensities of skin sensory stimulation in term infants. This indicates the potential for fMRI studies in exploring tactile and nociceptive processing in the infant brain.

**Key notes**

- This study used functional magnetic resonance imaging (fMRI) to assess the feasibility of measuring brain activation to a range of mechanical stimuli applied to the skin of neonatal infants.
- The results showed that somatosensory brain activation at term increases with stimulus strength and is reduced by sedation.
- Our findings demonstrate the feasibility of using fMRI to explore tactile and nociceptive processing in the infant brain.

## Introduction

The newborn human infant is almost continuously exposed to mechanical skin stimulation, whether it is through maternal contact, wrapping or spontaneous twitching and there is increasing evidence that this tactile input plays an important role in the growth and development of the brain (1,2). Furthermore, the excessive handling and skin breaking procedures experienced by preterm infants in neonatal intensive care are correlated with delayed cortical development (3). Despite this, little is currently known about somatosensory processing in the human infant brain or the pattern of activation in cortical and subcortical regions evoked by tactile and noxious stimulation of the newborn body surface.

Electromyographic measurement of lower limb flexion reflex responses to defined clinically required tactile and noxious stimuli show that preterm infants are very sensitive to cutaneous stimulation and display prolonged noxious evoked responses which gradually decrease with gestational age (4). This reflex activity, along with observed behaviours such as facial expressions and crying, and physiological responses, such as increased heart rate variability (5) confirm functional links between the somatosensory system and the motor and autonomic nervous system in even the most premature infants, but shed little light on how this information is processed at higher subcortical and cortical levels.

Lateralised haemodynamic activity in the newborn human infant somatosensory cortex has been recorded in response to noxious stimuli using near-infrared spectroscopy (6) and somatosensory (7), tactile and nociceptive event related potentials have been identified using time-locked electroencephalogram recording in neonates (8,9). These specific somatosensory cortical evoked potentials are evidence of the existence of neonatal brain circuits for touch and pain, emerging from immature nonspecific, evenly dispersed neuronal bursts at around 35 weeks of gestational age (8). Despite enabling high resolution in the time domain, near-infrared spectroscopy and electroencephalography do not provide direct information about localisation of somatosensory activity in the brain, particularly in subcortical brain structures. Functional magnetic resonance imaging (fMRI) is a noninvasive technique with high spatial resolution that allows visualisation of the cortical and subcortical structures involved in the response to a stimulus. In contrast to numerous studies on human adult somatosensory processing (10,11), very few fMRI studies have been performed on human infants. Ethical considerations and lack of verbal communication are a barrier, but do not preclude such studies in neonates (12). Previous studies have reported successful fMRI data acquisition in preterm and term infants under chloral hydrate sedation, in response to passive movement of the limb (13) or to passive flexion and extension of the hand (14,15). These predominantly proprioceptive sensory stimuli cause a clear area of activation in the contralateral primary somatosensory cortex, the supplementary motor area and the cerebellum (15) and less lateralisation than in adults (14). In addition, resting state network analysis in the preterm infant brain has identified the presence of the five main brain network patterns described in adults (16), which strengthen with gestational age from 29 to 43 weeks (17). However, a larger proportion of total brain activity lies in primary sensory and motor regions in infants, compared to the dominant association cortex activity in adults (18).

The aim of our research was to establish the feasibility of studying newborn brain activation to specific cutaneous sensory stimuli applied to a discrete area of the skin surface. Such studies could, in the future, be used to assess the extent to which infant cutaneous sensitivity, hyperalgesia and allodynia are processed at the cortical level.

## Methods

### Participants

A total of nineteen term born infants, aged 13 ± 10.5 (±SD) days, recruited from outpatients or inpatients at University College London Hospital, participated in the study (Tables1 and 2). Of these, 18 were scanned for clinical reasons, but were subsequently shown to have a normal radiological outcome. These eighteen infants had a mild hypoxic event at birth or an antenatal structural brain malformation, but the MRI scan at the time of study was reported as not presenting abnormal signal intensity in the brain parenchyma, especially the basal ganglia and the cortex, or any abnormal structural changes. Scans were reported for clinical purposes by a neuroradiologist and were later classified for this study by a neonatal brain expert. Images that suggested mild haematoma or bleeding remote from regions of interest were included, as these conditions were not expected to affect brain function. Exclusion criteria included infants in an unstable condition, which made additional scanning time impossible, and infants with lower limb deformations. Clinicians decided whether an infant was suitable for recruitment and for the study at the time of scan. Most infants had undergone therapeutic hypothermia at birth. No infants required ventilation or low flow oxygen. One infant was a healthy full-term infant scanned for research purposes only.

**Table 1:** Demographic characterisation of infant participants

Demographic characterisation of the sample	
Number of infants	19
Mean gestational age at birth (weeks)	40.0 (1.8); 37.1–44.1
Mean gestational age at study (weeks)	41.8 (2.2); 38.6–46.3
Mean postnatal age at study (days)	13 (10.5); 4–41
Number of male infants	11
Number of multiple gestation infants	0
Number of infants sedated	11
KEY: mean (standard deviation); range	

**Table 2:** Detailed characteristics of infant participants

Infant number	GA at birth (weeks)	GA at study (weeks)	PNA (days)	Sex	Sedation	Medication on day of study	Epochs accepted (max 11)	Stimulation	Reason for MRI	Structural MRI report at time of study
1	37.6	38.9	9	Female	Chloral hydrate	None	6	Brush	Mild neonatal encephalopathy*	Normal
2	38.4	39.0	4	Female	None	None	6	Brush	Mild neonatal encephalopathy*	Normal
3	40.9	42.0	8	Female	Chloral hydrate	None	8	Brush	Mild neonatal encephalopathy*	Normal
4	41.0	43.0	14	Female	Chloral hydrate	Antibiotics	6	Brush	Mild neonatal encephalopathy*	Normal
5	37.7	39.7	14	Male	None	None	10	Brush	Mild neonatal encephalopathy*	Normal
6	37.1	38.6	10	Male	None	None	9	Brush	Mild neonatal encephalopathy*	Normal
7	39.0	39.9	6	Female	Chloral hydrate	Antibiotics Food supplements	2	Brush	Mild neonatal encephalopathy*	Normal
8	41.7	43.0	9	Male	Chloral hydrate	None	5	Brush	Mild neonatal encephalopathy*	Normal
9	41.6	42.9	9	Female	Chloral hydrate	None	9 10	Brush and Punctate	Mild neonatal encephalopathy*	Normal
10	40.3	41.1	6	Male	Chloral hydrate	Antibiotics	4 6	Brush and Punctate	Mild neonatal encephalopathy*	Normal
11	40.6	41.1	4	Male	Chloral hydrate	None	4 5	Brush and Punctate	Mild neonatal encephalopathy*	Normal
12	41.9	42.7	6	Male	None	None	4 8	Brush and Punctate	Seizures and sepsis	Normal
13	41.4	46.3	34	Male	None	None	4 6	Brush and Punctate	Antenatal ventriculomegaly	Normal
14	39.3	40.7	10	Male	Chloral hydrate	None	9 8	Brush and Punctate	Mild neonatal encephalopathy*	Normal
15	39.7	45.6	41	Male	Chloral hydrate	None	6 8	Brush and Punctate	Mild neonatal encephalopathy	Normal
16	40.1	42.0	13	Male	Chloral hydrate	None	7 4	Brush and Punctate	Antenatal ventriculomegaly*	Normal
17	39.0	40.1	8	Male	None	None	8 5	Brush and Punctate	No clinical reason	Normal
18	39.0	43.1	29	Female	None	None	4	Punctate	Mild neonatal encephalopathy*	Normal
19	44.1	44.9	5	Male	None	None	7 5	Punctate and nondamaging pinprick	Poor respiratory effort at birth	Normal

GA = Gestational age.

* Hypothermia within 12 h of birth.

Ethical approval was obtained from both University College London and University College London Hospital Committees on the Ethics of Human Research. Informed written consent from the parent(s) or legal guardian was obtained prior to each study. The studies conformed to the standards set by The Declaration of Helsinki.

### Experimental protocol

Infants were prepared with ear protectors and physiological monitors and then swaddled in blankets with one foot exposed. The need for sedation was assessed by clinicians and was given as a 50 mg/kg bolus of chloral hydrate in 11 of 19 infants. All infants had been fed and were asleep before they were transferred in a supine position into a specially designed pod, which provided support and noise exclusion while they were inside the scanner.

During the fMRI acquisition the sole of the exposed foot was stimulated with a brush, a set of von Frey hairs and in one case, nontissue damaging pinprick. The brush stimulus was a SENSELab Brush no. 5 (0.5 g, 200–400 mN) from a standard quantitative sensory testing kit (Somedic, Hörby, Sweden) normally used to diagnose allodynia in adult neuropathic pain patients. The sole was brushed between the heel and the toe in stimulation blocks consisting of repeated, alternating up and down brushes with a stroke-length of 4–6 cm and a velocity of 7–9 cm/sec. Each block lasted 8 sec. The brush force did not result in passive movement.

Punctate stimulation was conducted with three intensities of nylon von Frey hair monofilaments (SenseLab Anesthesiometer; Somedic): 3.14, 10.8 and 50.0 mN. Each stimulus was applied to the plantar surface of the foot for 5 sec, with 1-sec from initial touch to buckling, 3 sec with a constant pressure and 1 sec to remove the filament. Stimuli were applied in a controlled order, each intensity preceded by varied intensities, so that preceding force was unlikely to affect the response to the subsequent force.

The nontissue damaging pinprick stimulation (MRC Systems, Heidelberg, Germany) was a 64 mN sliding barrel prick applied for 2 sec. All stimuli were tested by the parents on themselves as part of the consent procedure. In adults, low forces of vFh punctate and pinprick stimulation can activate A-δ nociceptors below pain intensities, producing a sensation of sharpness. None of the stimuli broke the surface of the skin or left any marks.

**Figure 1:**
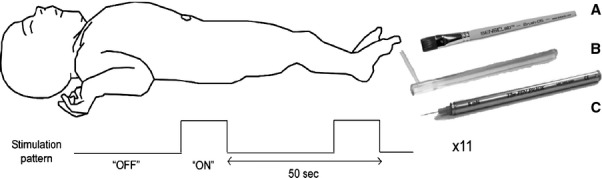
Stimulation protocol. The infant was supine with one foot exposed. The sole of the foot was stimulated with a (A) brush, (B) von Frey hair or (C) pinprick using a box-car paradigm of interblock interval of 42-50 sec.

Stimulation was applied using a box-car paradigm, eleven blocks of stimulation in total. The stimulation block duration was 8 sec for brush, 5 sec for vFh and 2 sec for nontissue damaging pinprick and hence the interblock stimulus interval was 42, 45 and 48 sec, respectively. The total scan time for the entire stimulation protocol in each infant was under 12 min. Figure1 illustrates the experimental protocol. All stimuli were applied by the same researcher (GW), and all timings recorded by the same research nurse (DJ). Data were event marked *post hoc* to determine blocks of stimulation.

### MRI acquisition and analysis

The scanner was a Siemens Avanto 1.5 Tesla with a knee coil. Structural scans and clinically required images were always acquired first. For details of the acquisition parameters, structural resolution and analysis of image data, see Supporting information Methods (Data S1).

## Results

### Distinct functional brain activation by cutaneous brush and vFh punctate stimulation in full-term infants

We administered the cutaneous brush stimulus to seventeen infants and eleven received vFh punctate stimuli, with nine of these infants receiving both (see Tables1 and 2). Cutaneous brush and punctate von Frey hair (vFh) stimulation of the plantar surface of the foot produced a significant positive blood-oxygen-level dependent (BOLD) response in every full-term infant tested. There was no significant negative BOLD on average whole brain analyses in response to any stimulus. An average of 6 ± 2.4 (SD) epochs (range 2–10) was accepted for each infant.

**Figure 2:**
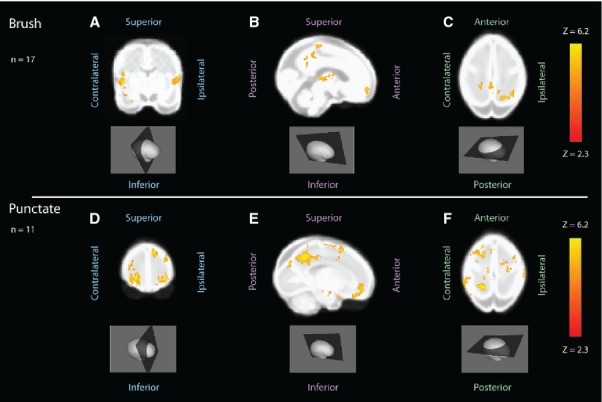
BOLD activation of the term infant brain following sensory stimulation of the plantar foot. (A–C) Group analysis of all accepted brush epochs in healthy term infants (n = 17), shown on a standard infant template brain (n = 17). Coronal view (A) shows positive BOLD activation in the insula. Sagittal view (B) shows positive BOLD at the midline in the primary somatosensory cortex, precuneus, thalamus, occipital and frontal cortex, and axial view (C) also shows positive BOLD in the primary somatosensory cortex. The coordinates of the three view planes are x:59 (coronal); y:87 (sagittal); z:80 (axial). (D–F) Group analysis of all accepted vFh punctate epochs in healthy term infants (n = 11), shown on a standard infant template brain, (n = 11). Coronal view (D) shows positive bilateral BOLD activation in the frontal cortex. Sagittal view (E) shows positive BOLD on the contralateral side in the precuneus, supplementary motor area, subgenual anterior cingulate cortex and frontal cortex, and axial view (F) also shows positive BOLD in the precuneus and supplementary motor area, frontal and parietal cortices. The coordinates of the three view planes are x:49 (coronal); y:114 (sagittal); z:84 (axial).

The Z-stat image resulting from the higher level analysis of all the accepted epochs (see Table2) shows that brushing the foot produces bilateral positive BOLD activation in SI, the precuneus, the medial frontal pole and the insula (Fig.2A–C). VFh punctate stimulation of the foot (combined data from all three hair forces) produces diffuse bilateral positive activation in the precuneus and the ventrolateral prefrontal cortex and in the supplementary motor area, subgenual cingulate cortex and dorsomedial prefrontal cortex (Fig.2D–F).

### Sedation reduces brain activation to von Frey hair punctate stimulation in full-term infants

Region of interest (ROI) analysis was undertaken to extract the activity in nine key areas of somatosensory activity, namely the ipsilateral and contralateral primary and secondary somatosensory cortices the ipsilateral and contralateral thalamus, the anterior cingulate and the ipsilateral and contralateral insula. As infants are frequently sedated for fMRI studies (19), we used ROI analysis to test the effect of sedation upon cutaneous sensory activation.

**Figure 3:**
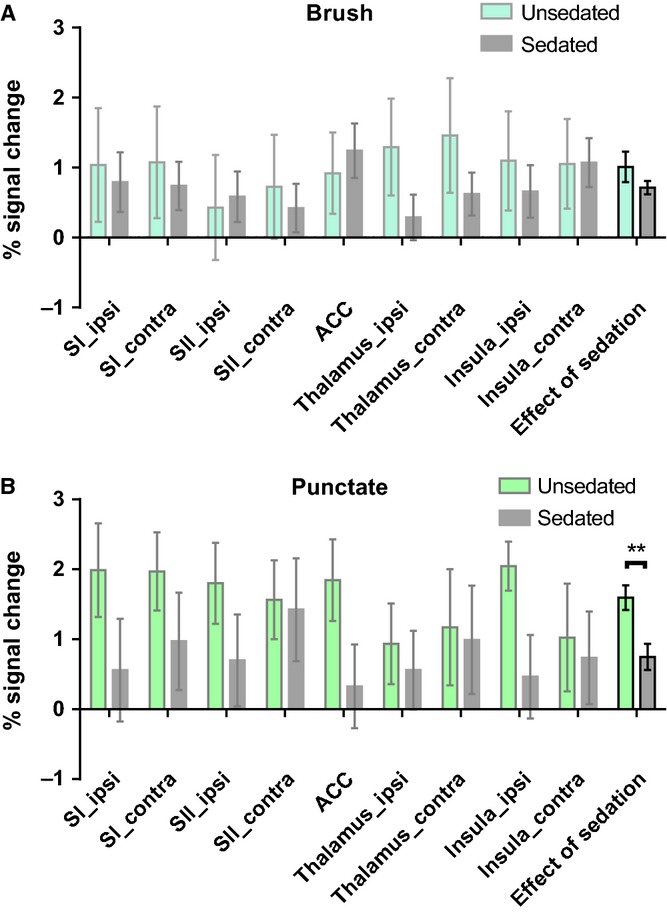
Mean percentage signal changes in unsedated and sedated infants in each region of interest in response to (A) brush stimulation (n = 6, 11) and (B) vFh punctate stimulation (n = 5, 6). Two-way ANOVA, represented by two bars on the far right, shows a significant effect of sedation upon activation by punctate stimulation (**p = 0.001), but not by brush stimulation. SI_ipsi = ipsilateral primary somatosensory cortex; SI_contra = contralateral primary somatosensory cortex; SII_ipsi = ipsilateral secondary somatosensory cortex; SII_contra = contralateral secondary somatosensory cortex; thalamus_ipsi = ipsilateral thalamus; thalamus_contra = contralateral thalamus; ACC = anterior cingulate cortex; insula_ipsi = ipsilateral insula; insula_contra = contralateral insula.

Figure3A shows the effect of sedation upon brush evoked activation in all nine ROIs and reveals a trend for reduced activation in many ROIs in sedated infants (n = 11) versus unsedated infants (n = 6). However, a two-way ANOVA revealed no significant effect of sedation upon brush activation.

The same analysis was performed on vFh punctate stimulation activation, using pooled data from all vFh intensities. Figure3B shows the effect of sedation upon vFh punctate activation in nine ROIs. Comparison of vFh punctate brain activation in sedated (n = 6) and unsedated (n = 5) infants across all ROIs revealed a highly significant effect of sedation upon vFh punctate activation (two-way ANOVA, p = 0.0078).

### Brain activation increases with cutaneous stimulus intensity

**Figure 4:**
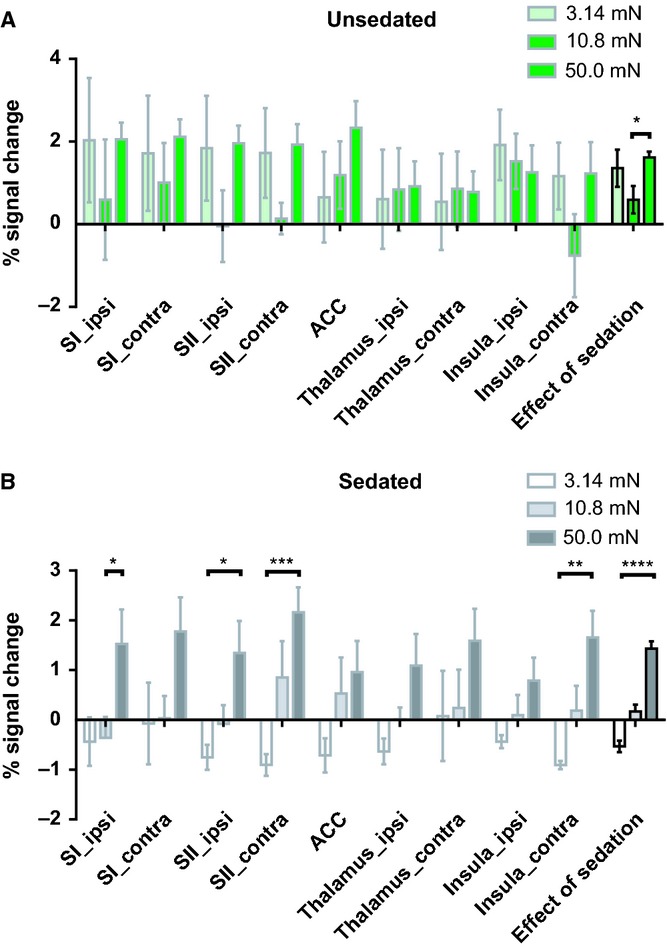
Mean percentage signal changes across three intensities of vFh punctate stimulation, 3.14, 10.8 and 50.0 mN, in each region of interest in (A) unsedated (n = 5) and (B) sedated (n = 6) infants. Two-way ANOVA shows significant changes in activation with increasing stimulus intensity in both groups (*p = 0.033 and ****p < 0.0001, represented by the three bars on the far right). In sedated infants, there were also significant changes with intensity in specific ROIs: ipsi SI (p < 0.05), ipsi SII (p < 0.05), contra SII (p < 0.001) and contra insula (p < 0.01). SI_ipsi = ipsilateral primary somatosensory cortex; SI_contra = contralateral primary somatosensory cortex; SII_ipsi = ipsilateral secondary somatosensory cortex; SII_contra = contralateral secondary somatosensory cortex; thalamus_ipsi = ipsilateral thalamus; thalamus_contra = contralateral thalamus; ACC = anterior cingulate cortex; insula_ipsi = ipsilateral insula; insula_contra = contralateral insula.

To test whether increasing cutaneous stimulus intensity increases brain activation in full-term infants, the activation was measured in response to three intensities of vFh punctate stimulation 3.14, 10.8 and 50.0 mN. All accepted epochs for each intensity for an infant were pooled. Figure4A shows the mean activation in each ROI for the three different vFh punctate stimulation forces in unsedated infants (n = 5) and Figure4B in sedated infants (n = 6). In both groups, stimulus intensity had a significant effect upon brain activation (two-way ANOVA, p = 0.033 for unsedated and p < 0.0001 for sedated). Figure4B also shows significant increases in brain activation with stimulus increasing intensity in specific ROIs in sedated infants: the ipsilateral primary somatosensory cortex (p < 0.05), ipsilateral secondary somatosensory cortex (p < 0.05), contralateral secondary somatosensory cortex (p < 0.001) and contralateral insula (p < 0.01).

### Nociceptive activation in a healthy unsedated infant

**Figure 5:**
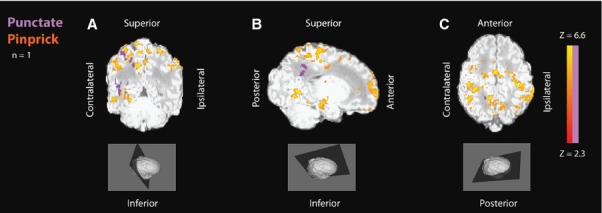
(A–C) Analysis of two accepted 50 mN punctate epochs (purple) and two accepted nontissue damaging pinprick epochs (red–yellow) from the same healthy term infant (infant 19), shown on the infant's own high-resolution structural MRI. Coronal view (A) shows bilateral positive BOLD activation particularly in the primary and secondary somatosensory cortices and the precuneus in response to nontissue damaging pinprick and a smaller areas of contralateral positive BOLD activation in the primary somatosensory cortex in response to vFh punctate stimulation. Sagittal view (B) shows positive BOLD activation in the contralateral side particularly in primary somatosensory cortex and the supplementary motor area, precuneus, occipital and frontal areas in response to nontissue damaging pinprick, and in the supplementary motor area and precuneus in response to vFh punctate stimulation. Axial view (C) shows positive BOLD activation particularly in the parietal cortices, which is much more widespread for nontissue damaging pinprick than for vFh punctate. The coordinates of the three view planes are x:48 (coronal); y:61 (sagittal); z:78 (axial).

To test the feasibility of using nontissue damaging cutaneous pinprick stimulation for fMRI studies in term infants, one unsedated healthy term infant, aged 5 days, received both vFh punctate and pinprick stimulation of the plantar surface of the foot. Figure5 shows that positive BOLD activation was more widespread in response to nontissue damaging pinprick compared to 50.0 mN vFh punctate stimulation. Two epochs for each condition were used to provide comparable signal-to-noise ratio. Significant bilateral activation was found in several regions including the primary somatosensory cortex, the secondary somatosensory cortex, the precuneus and frontal area and the supplementary motor area following nontissue damaging pinprick. ROI analysis showed that activation in every ROI was greater following nontissue damaging pinprick compared to 50.0mN vFh punctate stimulation, and the overall mean activation across ROIs in this single infant was significantly greater for nontissue damaging pinprick than for 50.0 mN vFh punctate stimulation (p = 0.001, paired *t*-test).

## Discussion

This fMRI study establishes the feasibility of studying brain activity to brush, vFh punctate and nontissue damaging pinprick of the skin in healthy term infants. All three stimuli, applied to the foot, were found to evoke significant and different patterns of activation in the newborn infant brain. Thus, as long as ethical and clinical issues are carefully considered (12), such studies are feasible and productive. Chloral hydrate, a safe and effective sedative for infant MRI studies (19), was used in 11 of the 19 infants studied and while, not surprisingly, this depressed functional activity, and significant activation was still recorded under sedation. Brain activations were determined by inspection of thresholded Z-stat images overlaid onto the standard infant template brain. This structural template was derived from term infants born prematurely (20) and, while not ideal for our term population, provided an unbiased method of localising activations. Region*s* of interest (ROI) analysis, performed on regions chosen on the basis of previous studies in adults (10,21), also revealed local modality and intensity specific patterns of activation.

### Patterns of brush and vFh punctate activation in the full-term infant brain

Functional activation in response to brush stimulation was located in the primary somatosensory cortex, precuneus, contralateral insula, thalamus, frontal cortex and occipital cortex. This is similar, but not the same, as the pattern of brush activation in adults where the somatosensory cortex, the contralateral mid- and posterior insula, the temporoparietal junction and the ipsilateral cerebellum are activated (22). No activation in the temporoparietal junction or the cerebellum was observed here, perhaps due to immature connections but also to the fact that infants were asleep or sedated. Thalamocortical projections are known to have reached the infant cortex by term (23), but little is known about other cortical connections at this time. Brush activation in infants was in the appropriate somatotopic primary somatosensory cortical location for the foot, in the midline grey matter, posterior to the central sulcus and contralateral to the side of stimulation. The brush stimulus used on the sole of the foot in this study is likely to be mediated by fast conducting myelinated (A*β*) afferents and in adults A*β* activation of the somatosensory cortex is associated with sensory rather than emotional aspects of touch (24). Innocuous brushing and noxious heat in adults produce significant activation in the contralateral primary and secondary somatosensory cortex (25) and bilateral processing between homotopic somatosensory regions of the opposite hemispheres (26), which is consistent with the ROI results presented here and with previous infant studies (13–15). These data suggest that these circuits are active at birth.

The brush and punctate induced precuneus activation in infants is notable as it has been implicated in higher level cognitive processes in adults (27), but the extensive connections, high resting metabolic rate and links with autonomic activation makes interpretation of activation in this region difficult. In adults, the precuneus forms part of the default mode network and becomes deactivated in response to stimulation (27). Resting state analyses have shown that the default mode network in infants is primitive (18) and does not include the precuneus (17). The concept that default mode network deactivation in response to a task develops in parallel with cognitive functions has been discussed in previous studies (17).

Although there were modality-specific patterns of larger clusters of activation, the fMRI Z-stat images are predominantly shown for illustrative purposes. The ROI analysis provides a more quantitative measure for comparison of activations.

### Intensity coding of cutaneous sensory stimulation in the human infant brain

The ROI analysis also showed that brain activation increased with stimulus intensity of von Frey hairs suggesting an ability of the human infant brain to code stimulus intensity to cutaneous mechanical stimulation. This was especially clear in sedated infants. We propose that this is because the general depressive effect of sedation across the brain increases the signal-to-noise ratio in some brain regions following cutaneous stimulation, making intensity coding easier to detect. The lower forces of vFh used in this study are likely to stimulate A*β* fibre mechanosensitive afferents but the higher forces will activate A*δ*-fibre nociceptors and therefore activate some nociceptive networks. A direct comparison can be made with an adult where punctate intensity (of higher forces 64–512 mN) correlated with brain activity, particularly in SI and ACC (28). While data from all infants with repeated vFh stimulation were pooled for this small study, in future it would be interesting to study the interaction between sedation, ROI and modality in the future.

This study was not set-up to make direct contrasts between brush and punctate modalities because of variances in stimulus duration and attentional qualities; however, our data support differences in activation between the two modalities in key brain regions. Therefore, future studies designed to make direct comparisons between stimulus modalities and examine how these change with time and postnatal development will provide important information.

### Feasibility of fMRI analysis of nociceptive brain activity in infants

One healthy term infant underwent nontissue damaging pinprick stimulation. The average functional activations in this infant were diffuse and mainly in bilateral SI and SII, SMA, the precuneus and the frontal cortex. The signal-to-noise ratio was low in this analysis due to the small number of events included. However, activation was somatotopically localised in the somatosensory cortical area appropriate for foot representation and areas activated are similar to those activated by pinprick in adult fMRI studies (29). Pinprick stimulation elicits a larger force on a smaller surface area than vFh punctate stimulation and specifically activates A*δ*-fibre nociceptors and specific pain-related cortical activity (30). The results illustrate that this stimulation could be used in future studies to map nociceptive activation in the human infant brain.

## Strengths and Limitations

This study was limited by the small number of participants and its exploratory nature. Nevertheless, it illustrates the feasibility of imaging somatosensory and nociceptive processing in neonates further. As in all fMRI studies, safety, resolution and haemodynamic response function estimation are important considerations, but fMRI does have an advantage over near-infrared spectroscopy and electroencephalography (6–9) in localising sensory evoked activity to specific brain areas.

Other factors should be considered here when interpreting results. The cohort studied was heterogeneous in demographics and characteristics, although those with abnormalities likely to affect somatosensory processing were omitted. Due to limited access to infants when inside the scanner, sleep state could not be categorised into active sleep or quiet sleep.

For analysis, several epochs had to be discarded due to high levels of head movement as it was not hospital policy to stabilise the heads of infants during scans. Deletion of contaminated epochs was a necessary compromise to achieve the best data set for analysis. Furthermore, the areas selected for ROI analysis were chosen based on adult studies, which may not be directly translatable into areas for somatosensory processing in infants. Brain activations were mostly bilateral, but the decision was made to flip structural and functional images in the axial plane where infants were stimulated on the right, to preserve ipsilateral and contralateral activations, whilst sacrificing lateralised activations.

The study had several key strengths. It was undertaken with methods to reduce bias and enhance the validity of the result, by use of an independent standard brain and consistent ROI definitions. The level for acceptance of epochs was stringent with only those where movement was <0.2 mm included. The use of a mixed effects analysis means that results are likely to be consistent across the general population. Importantly, we have adapted conventional fMRI methods appropriately to produce physiologically plausible brain activations with a small number of heterogeneous subjects.

## Conclusion

In conclusion, we have shown that it is possible to map tactile and punctate and nontissue damaging pinprick activity in the human infant brain using fMRI. At birth, distinct patterns of modality and intensity specific somatosensory BOLD activation are evoked by skin stimulation. This proof of principle study shows that this approach has considerable potential in the future to explore the development of tactile and nociceptive processing in the preterm and term human brain.

## Abbreviations

ANOVA: Analysis of variance
BOLD: Blood-oxygen-level dependent
fMRI: Functional magnetic resonance imaging
mN: Millinewton
ROI: Region of interest
SD: Standard deviation
vFh: von Frey hair
