# Supplementary material for: Functional magnetic resonance imaging can be used to explore tactile and nociceptive processing in the infant brain
Source: Acta Paediatr. 2014 Dec 1;104(2):158–66. doi: 10.1111/apa.12848 (PMC4463763; doi:10.1111/apa.12848)
Supplement: Data S1 [file apa0104-0158-sd1.docx]

**Supplementary Methods:**

**MRI acquisition**

The scanner was a Siemens Avanto 1.5 Tesla with a knee coil. Structural scans and clinically required images were always acquired first. The high resolution structural image provided a reference for the functional image and was acquired with a T1-weighted 3D FLAIR sagittal sequence (acquisition parameters: (TR) 17 ms; (TE) 6.06 ms; (flip angle) 21°; (matrix) 256*200; (resolution (x∗y∗z)) 1∗1∗1 mm; (field of view) 100%). The functional images were acquired with a continuous interleaved echo-planar 2D sequence covering the whole brain (acquisition parameters: (TR) 3500 ms; (TE) 90 ms; (flip angle) 90^o^; (field of view) 100%; (resolution (x∗y∗z) 2∗2∗3 mm, total volumes 150).

### Whole brain analysis

Structural images were cropped, and Brain Extraction Tool, part of FSL (FMRIB Software Library, [www.fmrib.ox.ac.uk/fsl](http://www.fmrib.ox.ac.uk/fsl)), was used to segment and remove non-brain areas from the high-resolution structural image (1). Functional data were analysed using FEAT (FMRI Expert Analysis Tool) Version 5.98 which is part of FSL (2,3). Some volumes were contaminated with excessive motion artefact during functional acquisition which could not be reliably corrected for with FSL. In order to account for this, functional files were segmented into epochs composed of four volumes before the stimulation and nine volumes afterwards. First level analysis was then performed only for those epochs in which the detected relative head movement was less than 0.2mm to ensure that the data was not contaminated by movement artefact.

A high-pass temporal filter (a Gaussian weighted straight line with a 45.5 second cut off) was applied to remove low frequency drifts lasting the length of the epoch. The functional data were smoothed spatially with a Gaussian kernel with a full width at half-maximum of 2 mm. Motion correction was performed using Motion Correction - FMRIB’s Linear Image Registration Tool (MCFLIRT) (4). A general linear model was applied on a voxel by voxel basis using FMRIB’s improved linear model (FILM) to model blood oxygen level dependent (BOLD) signal intensity changes (5). Data was convolved to a haemodynamic response function (HRF) which peaked at 8 seconds appropriate for the gestational age at time of study (6). The duration of the stimulus was used to scale each HRF in time. Positive and negative contrasts were investigated.

Images were corrected for multiple spatial comparisons according to Gaussian random field theory (7). Statistical images were registered to the high-resolution structural image of the particular infant using FLIRT (8). Secondary registration was conducted to a template infant brain at the particular age of study of that infant, and then to a standard T1-weighted infant brain (9).

The results of the first level analyses conducted on the individual epochs were then combined in a higher level analysis with mixed effects using FLAME (FMRIB’s Local Analysis of Mixed Effects) 1+2 to create the average functional activations for each infant, and for each condition. If infants were stimulated on the right foot (n=5), functional and structural images were flipped in the axial plane. Z statistic images were corrected for multiple comparisons with an automated cluster-based correction based upon Gaussian random field theory, determined by Z > 2.3 thresholding and a cluster significance level defined according to spatial extent at p < 0.05. Anatomical regions of activation were identified by inspection of functional activation maps overlaid onto the T1-weighted standard infant brain.

### Region of Interest Analysis

Region of interest (ROI) analysis was used to perform a statistical comparison of activation in specific regions between different conditions, stimulus modalities and intensities. ROIs were selected according to reported importance in adult fMRI studies of tactile and nociceptive brain activation (10,11): primary somatosensory cortex (SI), secondary somatosensory cortex (SII), anterior cingulate cortex (ACC), thalamus and insula. For each structure, ipsilateral and contralateral areas were investigated separately except the ACC which was considered as a single area because of its proximity to the midline. There are no standardised labelled templates and atlases available for infant brains, such as the adult brain Montreal Neurological Institute template with the Talairach and Tournoux atlas (12–14). Therefore, ROIs were defined anatomically with reference to an infant brain atlas (15) and manually drawn over a T1-weighted template infant brain in FSLView, so that the ROIs were identical for all infants (9). Since this template was designed to segment cortex, white matter, deep grey matter, cerebellum, brainstem and cerebrospinal fluid, the fine structures of many relevant gyri and sulci were not preserved; thus definition of ROIs was necessarily approximate (see Fig.S1). However, this method was considered less biased than using a


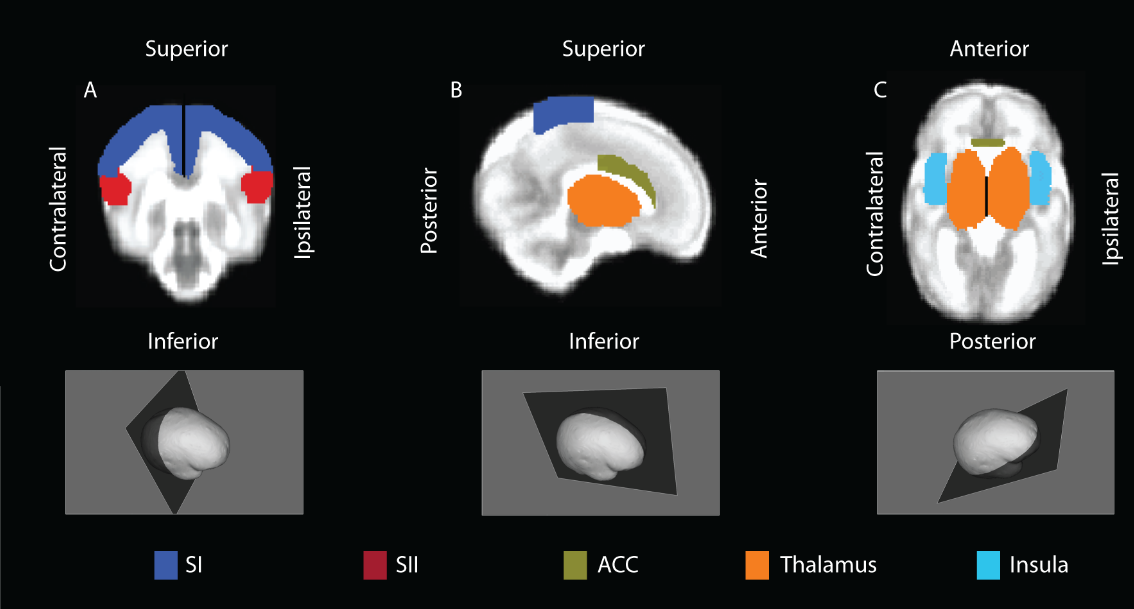


Fig.S1 Regions of interest shown on the standard template infant brain. For each region apart from ACC, ipsilateral and contralateral regions were investigated separately. Laterality is shown when necessary by a solid black line. Coordinates are x:64; y:67; z:56.’

high-definition structural image from a single full term infant (9). As the central sulcus was identifiable on the structural template, SI could be defined as the postcentral gyrus with careful reference to width using the infant brain atlas (15). The insula encompassed both anterior and posterior regions and was defined as cortical grey matter medial to the lateral cerebral fissures, running in the coronal plane. SII was defined with reference to the insula, as the cortical grey matter area which is superior to the insula and not on the external surface of the brain. The thalamus and ACC were defined according to relative local changes in density, for example the lateral edge of the thalamus was defined as the beginning of capsular white matter and lateral edge of the ACC as the beginning of the lateral ventricles.

A representative activation value for each ROI was calculated as the average signal change in the top 20% of the voxels with the highest absolute Z-score (regardless of whether they survived the threshold of Z > 2.3), and the mean percentage signal change in each area was compared across groups. Using the mean of the top 20% of active voxels has been shown to be more sensitive than using the mean of all voxels (16). The effects of sedation and stimulus intensity were tested using 2-way ANOVA, followed by Bonferroni multiple comparisons test.

**References**

1. Smith SM. Fast robust automated brain extraction. *Human Brain Mapping*. 2002;17(3):143–55.

2. Smith SM, Jenkinson M, Woolrich MW, Beckmann CF, Behrens TEJ, Johansen-Berg H, et al. Advances in functional and structural MR image analysis and implementation as FSL. *NeuroImage*. 2004;23, Supplement 1:S208–S219.

3. Woolrich MW, Jbabdi S, Patenaude B, Chappell M, Makni S, Behrens T, et al. Bayesian analysis of neuroimaging data in FSL. *Neuroimage*. 2009 Mar;45(1 Suppl):S173–186.

4. Jenkinson M, Bannister P, Brady M, Smith S. Improved optimization for the robust and accurate linear registration and motion correction of brain images. *Neuroimage*. 2002 Oct;17(2):825–41.

5. Woolrich MW, Ripley BD, Brady M, Smith SM. Temporal autocorrelation in univariate linear modeling of FMRI data. *Neuroimage*. 2001 Dec;14(6):1370–86.

6. Arichi T. Functional MRI of the developing neonatal brain: potential and challenges for the future. *Dev Med Child Neurol*. 2012 Aug;54(8):680.

7. Worsley KJ, Evans AC, Marrett S, Neelin P. A three-dimensional statistical analysis for CBF activation studies in human brain. *J Cereb Blood Flow Metab*. 1992 Nov;12(6):900–18.

8. Jenkinson M, Smith S. A global optimisation method for robust affine registration of brain images. *Med Image Anal*. 2001 Jun;5(2):143–56.

9. Kuklisova-Murgasova M, Aljabar P, Srinivasan L, Counsell SJ, Doria V, Serag A, et al. A dynamic 4D probabilistic atlas of the developing brain. *Neuroimage*. 2011 Feb 14;54(4):2750–63.

10. Apkarian AV, Bushnell MC, Treede R-D, Zubieta J-K. Human brain mechanisms of pain perception and regulation in health and disease. *Eur J Pain*. 2005 Aug;9(4):463–84.

11. Tracey I. Nociceptive processing in the human brain. *Curr Opin Neurobiol*. 2005 Aug;15(4):478–87.

12. Lancaster JL, Woldorff MG, Parsons LM, Liotti M, Freitas CS, Rainey L, et al. Automated Talairach atlas labels for functional brain mapping. *Hum Brain Mapp*. 2000 Jul;10(3):120–31.

13. Maldjian JA, Laurienti PJ, Kraft RA, Burdette JH. An automated method for neuroanatomic and cytoarchitectonic atlas-based interrogation of fMRI data sets. *Neuroimage*. 2003 Jul;19(3):1233–9.

14. Talairach J, Tournoux P. Co-planar stereotaxic atlas of the human brain. New York: Thieme; 1988.

15. Bayer S A, Altman J. Atlas of Human Central Nervous System Devlelopment: The Human Brain during the Third Trimester. CRC Press; 2003.

16. Mitsis GD, Iannetti GD, Smart TS, Tracey I, Wise RG. Regions of interest analysis in pharmacological fMRI: how do the definition criteria influence the inferred result? *Neuroimage*. 2008 Mar 1;40(1):121–32.
